# Mycorrhizal Inoculation Enhances Drought Tolerance in Potato (*Solanum tuberosum* L.) by Modulating Antioxidant Enzyme Activity and Related Gene Expression

**DOI:** 10.3390/biology15020180

**Published:** 2026-01-19

**Authors:** Souad Ettlili, Ricardo Aroca, Naceur Djebali, Sonia Labidi, Faysal Ben Jeddi

**Affiliations:** 1 Laboratory of Horticultural Sciences LR13AGR01, National Agronomic Institute of Tunisia, University of Carthage, 43 Av. Charles Nicolle, Tunis 1082, Tunisia; souad.ettlili@inat.ucar.tn (S.E.); sonia.labidi@inat.ucar.tn (S.L.); faysal.benjeddi@inat.ucar.tn (F.B.J.); 2 Department of Soil and Plant Microbiology, Zaidín Experimental Station, Spanish Research Council (CSIC), Prof. Albareda 1, 18008 Granada, Spain; 3 Laboratory of Bioactive Substances, Center of Biotechnology of Borj Cedria, BP 901, Hammam-Lif 2050, Tunisia; biotech@cbbc.rnrt.tn

**Keywords:** potato, mycorrhizal inocula, drought stress, antioxidant mechanism, super oxide dismutase, beneficial microorganisms, photosynthetic efficiency

## Abstract

Potato (*Solanum tuberosum* L.) is one of the most widely cultivated crops in the world; however, drought is a major constraint to its productivity. Arbuscular mycorrhizal fungi (AMF) have been shown to improve plant resistance under conditions of water stress. However, their effects on potato plants are poorly studied. The purpose of this study was to evaluate the potential of two AMF inocula (two different strains of the AMF species *Rhizophagus irregularis* with different origin: Southern Spain MI_1_ and Tunisia MI_2_) on potato tolerance to drought stress through the determination of growth parameters, photosynthetic parameters, and antioxidant systems, under well-watered (WW; field capacity) and drought stress (DS; 50% of field capacity) conditions. Therefore, the experiment consisted of two factors: AMF strain and watering regime. The results showed that under drought stress conditions, AMF inoculation considerably stimulated photosynthetic performance as compared with non-inoculated controls. Moreover, leaf superoxide dismutase (SOD) and catalase (CAT) activities of inoculated plants were higher in WW conditions, but unchanged in DS conditions. Inoculated plants had significantly higher ascorbate peroxidase (APX) and glutathione reductase (GR) activities than non-inoculated plants under DS conditions. Also, expression of some antioxidant enzyme genes were upregulated by inoculation. Lipid peroxidation content of inoculated plants was lower than that of non-inoculated. Furthermore, there was a high positive correlation between mycorrhizal root colonization (RC) and almost all the measured parameters. The results of this study indicated that AMF inoculation could enhance potato plant tolerance to water stress through the induction of antioxidant mechanisms implicated in scavenging oxygen-free radicals.

## Simple Summary

Potato production is highly sensitive to drought, which threatens yields in many regions. This study examined whether beneficial soil fungi known as arbuscular mycorrhizal fungi can help potato plants better tolerate periods without enough water. We tested two types of fungal inocula and compared inoculated and non-inoculated plants grown under normal watering and drought. We evaluated plant growth, leaf pigments involved in photosynthesis, markers of cellular damage, and several natural defense systems that plants use to protect themselves from stress. The results showed that inoculated plants maintained better leaf function, kept more chlorophyll and carotenoids, and regulated water loss more effectively during drought. They also activated important protective systems that help the plant neutralize harmful particles produced under stress. As a result, inoculated plants suffered less cellular damage than non-inoculated ones. Overall, this study shows that these beneficial fungi can strengthen potato plants and help them better withstand drought, offering a natural and environmentally friendly strategy to support crop production under water-limited conditions.

## 1. Introduction 

Potatoes (*S. tuberosum*) are among the world’s most widely cultivated crops, making a significant contribution to the global food supply [1]. As a staple crop, potatoes are essential to the diets of millions of people and serve as a critical source of starchy carbohydrates, proteins, essential amino acids, minerals, antioxidants, and vitamins [2,3,4]. Globally, approximately 380 million tons of potato tubers are produced annually, with 20 million hectares devoted to potato cultivation [5]. However, potato production faces significant constraints due to its vulnerability to environmental stresses, particularly drought.

Drought can severely impair potato growth and productivity, resulting in substantial losses in both yield and tuber quality [6,7]. Its negative effects are manifold, affecting physiological processes such as photosynthesis, stomatal conductance, and overall biomass accumulation [8]. Drought reduces photosynthetic efficiency through stomatal closure, limiting carbon dioxide availability in the leaf mesophyll [9]. Consequently, this decrease in photosynthetic activity can adversely impact tuber growth and development [7].

Water stress also leads to the accumulation of reactive oxygen species (ROS), including superoxide (O_2_^−^), singlet oxygen (^1^O_2_), hydroxyl radicals (OH^−^), and hydrogen peroxide (H_2_O_2_), which can cause significant oxidative damage to cellular components [10,11]. ROS accumulation disrupts the integrity of cellular organelles and impairs physiological functions. In response, plants activate antioxidant defense mechanisms composed of both enzymatic and non-enzymatic components [12]. Among non-enzymatic agents, ascorbic acid (AsA) and glutathione (GSH) play key roles, while enzymatic defenses involve the regulation of superoxide dismutase (SOD), peroxidase (POD), and catalase (CAT) activities [13,14]. These enzymes are crucial for scavenging excessive ROS, protecting metabolic processes, and maintaining cellular homeostasis [15].

Numerous studies emphasize the importance of balancing ROS production and scavenging to prevent cellular damage and enhance plant resilience under drought [16,17,18]. Maintaining this balance is essential for preserving photosynthetic machinery, membrane stability, and overall growth and productivity under environmental stress [19].

Given these challenges, developing sustainable strategies to mitigate drought effects on crops is critical. Among these, inoculation with beneficial microorganisms such as arbuscular mycorrhizal fungi (AMF) has emerged as a promising approach to enhance drought tolerance [20]. AMF form symbiotic associations with plant roots, improving water and nutrient uptake [21,22] and influencing various physiological and biochemical processes, thereby increasing drought resilience [23]. Moreover, AMF exude a glycoprotein called glomalin into the soil, which improves soil structure and enhances water retention [24].

Recent studies show that AMF inoculation can enhance the activity and gene expression of antioxidant enzymes, improving drought tolerance in several crops [25]. For example, He et al. [26] reported that maize plants inoculated with AMF exhibited higher SOD, CAT, and POD activities under drought compared to non-inoculated plants. Similarly, mycorrhizal inoculation in tomato has been shown to upregulate genes encoding antioxidant enzymes, enhancing drought resilience [27]. However, the role of AMF in modulating the antioxidant defense system under drought in potatoes remains poorly understood.

This study aims to investigate the effect of AMF inoculation on drought tolerance in potato by assessing the activity and gene expression of key antioxidant enzymes. Considering that the response to AMF is strain-dependent [28], two distinct AMF strains were used to explore potential differences in plant response. Understanding these mechanisms may inform the application of mycorrhizal biotechnology as a sustainable strategy to mitigate drought effects on potato production. The novelty of this research lies in evaluating the effects of different AMF strains on drought tolerance at the leaf level—a less extensively studied aspect of plant–AMF interactions. The findings are expected to contribute to improving agricultural practices to enhance food security in the context of climate change.

## 2. Materials and Methods

### 2.1. Experimental Design

A pot experiment was conducted at the Zaidín Experimental Station, in the Soil and Plant Microbiology department (Mycorrhiza laboratory). It consisted in a randomized complete block design (since pots were used) with two main factors: (1) mycorrhizal inoculation, which included non-inoculated control plants (C), plants inoculated with the AM fungus *Rhizophagus irregularis*, reproduced at the collection of the Zaidin Experimental Station (isolate EEZ 58) (MI)_1_ and plants inoculated with *R. irregularis* strain, provided by the Horticultural Sciences Laboratory of the National Agronomic Institute of Tunisia (LSH-INAT) (MI)_2_ and (2) water irrigation, where one half of the plants were grown under well-watered (WW) conditions during the entire experiment, while the other half of plants were subjected to drought stress (DS), 21 days after planting (when 50% of the plants at least reached the 4 leaves stage). This resulted in six treatments: well-watered non-inoculated plants (C-WW), inoculated plants with isolate EEZ 58 well-watered (MI_1_-WW), inoculated plants with Tunisian isolate well-watered (MI_2_-WW), non-inoculated plants subjected to drought (C-DS), inoculated plants with isolate EEZ 58 subjected to drought (MI_1_-DS), and inoculated plants with Tunisian isolate subjected to drought (MI_2_-DS). Each treatment had 11 replicates.

### 2.2. Soil and Biological Materials

The soil used in this study was collected from IFAPA grounds (Granada, Spain) on 20 September 2022. It underwent a sieving process (2 mm) and it was mixed with quartz sand (<1 mm) at a ratio of 1:3 (soil: sand, *v*/*v*), and subsequently subjected to sterilization through steam treatment (100 °C for 1 h on 3 consecutive days). The original soil had a pH of 8.1 [measured in water 1:5 (*w*/*v*)], and it contained 0.85% organic matter, along with the following nutrient concentrations (mg/kg): N, 1; P, 10 (NaHCO_3_-extractable P); and K, 110. The composition of the soil was determined and it comprises 38.3% sand, 47.1% silt, and 14.6% clay.

Tubers of *S. tuberosum* cultivar Colomba, were purchased from the supermarket with a medium caliber (diameter ranging between 40 and 55 mm). Tubers were incubated at room temperature for 21 days and then transferred to pots of 5 L capacity containing 4 Kg of the soil/sand mixture described above. Sixty-six pots were prepared, each one containing one potato sprouted tuber.

The mycorrhizal inoculum contained a mixture of soil, spores, mycelia, and infected root fragments of sorghum growing plants. The AM fungus was *R. irregularis*, belonging to the EEZ Collection, strain EEZ 58 (MI)_1_ and the LSH-INAT collection (MI)_2_. Five grams of inoculum, containing approximately 650 infective propagules per gram (as determined by the most probable number test), were placed into the chosen pots during the transplantation of tubers. Non-inoculated control plants were treated with a 10 mL aliquot of a <20 µm filtrate obtained from both AM inocula to establish a complete microbial community free of AM propagules.

### 2.3. Growth Conditions

The experiment was carried out under glasshouse conditions with temperatures ranging from 18 to 25 °C, 16/8 light/dark period, and a relative humidity of 50–60%. Plants were cultivated for a total of 8 weeks, and 3 weeks after planting, water-stressed plants started receiving half of the quantity of water per pot. All pots were weighed twice a week to determine the water quantity to add. Soil water field capacity was calculated as described by Sánchez-Romera et al. [29]. Well-watered plants were watered up to field capacity twice a week, while drought plants were watered up to 50% of field capacity twice a week too. Additionally, all plants were normally fertilized (2.17, 1.63 and 3.91 g/pot of N, P_2_O_5_ and K_2_O, respectively). Tap water was used for watering.

### 2.4. Measurements

#### 2.4.1. Shoot Biomass Production and Symbiotic Development

Upon harvesting, the plant shoots were weighed in order to ascertain their fresh weights (FW). Subsequently, the dry weight (DW) was also determined after drying in a forced hot-air oven at 70 °C for 2 days.

The mycorrhizal root colonization’s percentage in potato plants was determined through microscopic examination of fungal colonization. This process involved cleaning the washed roots in a 10% KOH, followed by staining with 0.05% trypan blue in lactic acid (*v*/*v*), in accordance with the methodology established by Phillips and Hyman [30]. The rate of mycorrhizal colonization was assessed through the gridline intersect method [31].

#### 2.4.2. Efficiency of Photosystem II

The photosystem II efficiency was assessed using a FluorPen FP100 (Photon Systems Instruments, Brno, Czech Republic), enabling non-invasive evaluation of plant photosynthetic performance by measuring chlorophyll a fluorescence. For each treatment, measurements were taken one day before harvest on the second youngest, fully expanded leaf, taken in a fully sunlight position. The measurements were taken in the 11 plants per treatment.

#### 2.4.3. Stomatal Conductance

Stomatal conductance (gs, mmol H_2_O m^−2^ s^−1^) was measured two hours after light turned on by using a porometer system (Porometer AP4, Delta-T Devices Ltd., Cambridge, UK) following the user manual instructions. For each treatment, measurements were taken one day before harvest on the second youngest, fully expanded leaf, taken in a fully sunlight position.

#### 2.4.4. Chlorophylls and Carotenoids Contents

Leaf samples were crushed with liquid nitrogen and 100 mg of the sample was weighed, mixed with 1 mL of methanol, 100% (pure solvent), stirred, and finally incubated in darkness at 4 °C for 24 h. Afterward, the samples were centrifuged at 10,000 rpm at 4 °C for 5 min. Chlorophyll a (Chla), chlorophyll b (Chlb), total chlorophylls (Chl-tot), and carotenoids (Car) contents were determined using a microplate spectrophotometer (Infnite M200 NanoQuant, Tecan, Zurich, Switzerland) at 470, 652.4, and 665.2 nm. The pigment concentrations were determined according to formula developed by [32].

#### 2.4.5. Malondialdehyde Content (MDA)

The measurement of malondialdehyde (MDA) contents was conducted spectrophotometrically through a modified approach based on the technique outlined by [33]. Potato leaves samples from each treatment (100 mg) were homogenized in 1.5 mL of 80% ethanol (*v*/*v*) and centrifuged at 3000× *g* for 10 min at 4 °C. The supernatant obtained was combined with 700 µL of 20% TCA (trichloroacetic acid, *v*/*v*) that included 0.5% TBA (2-thiobarbituric acid) and subsequently heated at 95 °C in a block heater for 25 min. After heating, it was quickly cooled in an ice bath. The samples underwent a second centrifugation at 3000× *g* for a duration of 10 min. The concentration of MDA was subsequently measured using a spectrophotometer and calculated based on the differences in absorbance at 440, 532, and 600 nm, following the formula referenced by [34].

#### 2.4.6. Antioxidant Enzyme Activities

The procedure outlined by [35] for enzyme extraction was followed. In a cooled mortar, 50 mg of polyvinylpolypyrrolidone (PVPP) and 5 mL of 100 mM phosphate buffer (pH 7.0) supplemented with 0.1 mM diethylenetriamine pentaacetic acid (DTPA) were combined with around 0.25 g of leaf tissue. By eliminating phenolic chemicals and alkaloids from the plant extracts, this formulation improved enzyme stability and reduced interference with spectrophotometric analyses. The extract was centrifuged for 10 min at 38,000× *g* after it had been filtered. The antioxidant enzymes’ activity was assessed using the supernatant. Superoxide dismutase (SOD), glutathione reductase (GR), and ascorbate peroxidase (APX) activity were assessed using the procedures outlined in Aroca et al. [35]. The enzymatic activity of catalase (CAT) was evaluated using the procedure described by Aebi [36], which consisted of monitoring the decrease in H_2_O_2_ at a wavelength of 240 nm for a period of 1 min, applying an extinction coefficient of 39.6 mM^−1^ cm^−1^. The reaction solution was formulated with 50 mM phosphate buffer at a pH of 7.0, 10 mM H_2_O_2_, and incorporated 100 µL of enzyme extract to achieve an overall volume of 2 mL.

#### 2.4.7. Expression Analysis of Antioxidant Genes

Three biological replicates of potato leaves were used for the extraction of total RNA, following the method outlined by Quiroga et al. [37]. The synthesis of first-strand cDNA was accomplished using random hexamers as primers alongside SuperScript-II RT (Invitrogen, Carlsbad, CA, USA). The amplification of antioxidant enzymes genes (SOD, CAT, APX, and GR) through real-time PCR was carried out with the CFX96 Touch Real-Time PCR Detection System (Bio-Rad, Hercules, CA, USA) employing gene-specific primers indicated in Table 1. These primers were chosen as described previously by [10,38]. The PCR was conducted using the SYBR Green PCR kit (Qiagen, Hilden, Germany), with actin serving as the internal control. The PCR settings included an initial denaturation phase at 95 °C lasting 5 min, followed by 30 cycles consisting of denaturation at 94 °C for 30 s, annealing for 30 s at 50 °C, followed by 2 min of extension at 72 °C. Threshold (Ct) values were normalized against the actin control and applied to ascertain the relative levels of gene expression.

### 2.5. Statistical Analysis

For statistical analysis, R software (v.4.3.1) was used. The effect of two factors, AMF inoculation and water irrigation regimes, and their interactions on measured parameters was performed using the two-way analysis of variance (ANOVA). A post hoc analysis was carried out using Tukey’s multiple comparison test. Correlation between measured variables was assessed using Pearson’s correlation coefficient (*p* < 0.05). The principal component analysis (PCA) was performed using the FactoMineR [39] and Factoextra [40] R libraries.

## 3. Results

### 3.1. Root Mycorrhizal Colonization

**Figure 1:**
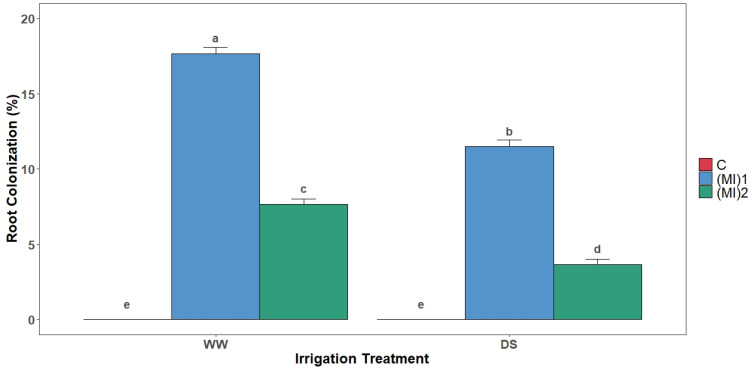
Root colonization rate of potato (*Solanum tuberosum* L.) plants non-inoculated (control: C) or inoculated with the two mycorrhizal inocula (MI)_1_ and (MI)_2_ and cultivated under well-watered (WW) and drought stress (DS) conditions. Different letters indicate significant differences between all treatments according to two-way ANOVA followed by Tukey’s test (*p* < 0.05).

The microscopic observations demonstrated that the roots of non-inoculated plants were not colonized by AMF. However, for the inoculated plants, mycorrhizal colonization varied between 3.3 and 17.5% for (MI)_2_ and (MI)_1_, respectively (Figure 1). The water stress conditions (DS) induced a significant lower mycorrhizal root colonization than the well-watered conditions (WW). In fact, DS generated a root colonization decrease of 38.3 and 51.2% for (MI)_1_ and (MI)_2_, respectively (Figure 1).

### 3.2. Shoot Fresh and Dry Biomass

**Figure 2:**
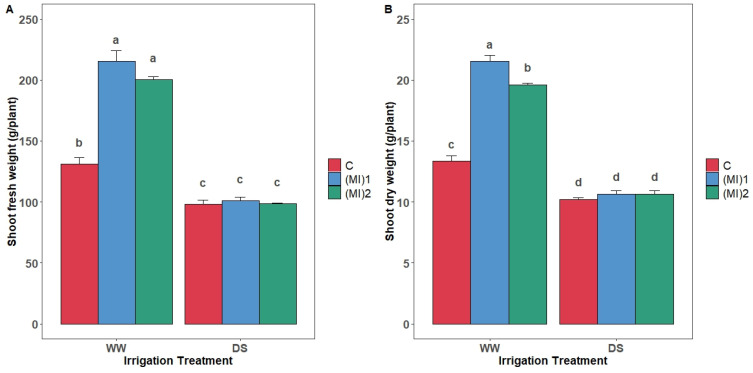
Shoot fresh biomass (**A**) and dry biomass (**B**) of potato (*Solanum tuberosum* L.) plants non-inoculated (control: C) or inoculated with the two mycorrhizal inocula (MI)_1_ and (MI)_2_ and cultivated under well-watered (WW) and drought stress (DS) conditions. Different letters indicate significant differences between all treatments according to two-way ANOVA followed by Tukey’s test (*p* < 0.05).

Under well-watered (WW) conditions, potato’s shoot fresh biomass was higher in inoculated plants compared to non-inoculated (Figure 2A). Under drought conditions, no significant difference was observed between the treatments. Furthermore, under WW conditions, the inoculation with AMF significantly increased the shoot dry biomass by 61.4 and 47.2% for (MI)_1_ and (MI)_2_, respectively, compared with non-inoculated plants (Figure 2B). No significant difference was observed between the dry biomass of control and inoculated plants under water stress conditions.

### 3.3. Photosynthetic Efficiency and Stomatal Conductance

**Figure 3:**
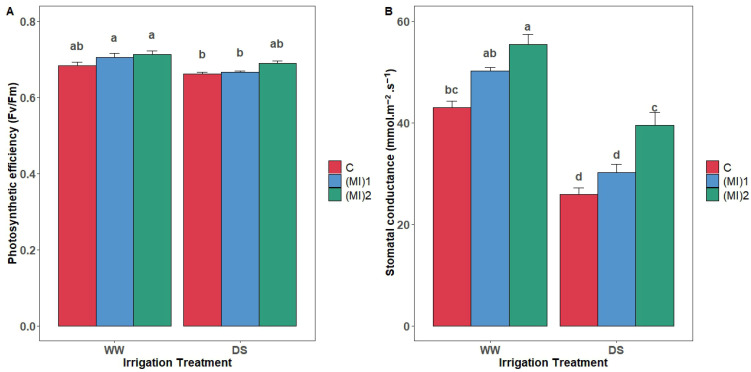
Photosynthetic efficiency (**A**) and stomatal conductance (**B**) of potato (*Solanum tuberosum* L.) plants non-inoculated (control: C) or inoculated with the two mycorrhizal inocula (MI)_1_ and (MI)_2_ and cultivated under well-watered (WW) and drought stress (DS) conditions. Different letters indicate significant differences between all treatments according to two-way ANOVA followed by Tukey’s test (*p* < 0.05).

The highest values of photosynthetic efficiency (PSE) were observed under well-watered conditions (Figure 3A). Plants inoculated with AMF and grown under well-watered conditions showed slightly higher values than the control. Furthermore, for water stress conditions, PSE was marginally affected in plants inoculated with the mycorrhizal inoculum (MI)_2_.

The positive effect of mycorrhizal inoculation on gs was more pronounced under drought conditions (Figure 3B). In fact, plants inoculated with (MI)_2_ reported the highest stomatal conductance value compared to the control, with an increase of 52.5%, such difference being statistically significant (*p* < 0.05).

### 3.4. Photosynthetic Pigments

**Figure 4:**
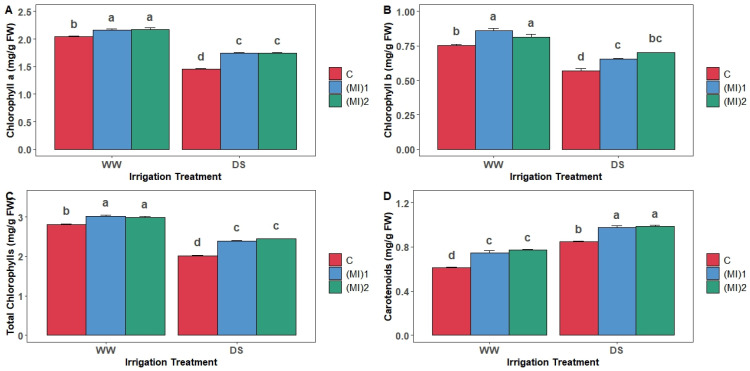
Chlorophyll a (**A**), chlorophyll b (**B**), total chlorophylls (**C**), and carotenoids (**D**) contents in leaves of potato (*Solanum tuberosum* L.) plants non-inoculated (control: C) or inoculated with the two mycorrhizal inocula (MI)_1_ and (MI)_2_ and cultivated under well-watered (WW) and drought stress (DS) conditions. Different letters indicate significant differences between all treatments according to two-way ANOVA followed by Tukey’s test (*p* < 0.05).

Chlorophylls and carotenoids contents were increased by AMF application under well-watered conditions, but it was affected under conditions of water deficit (Figure 4A–C). However, this decrease was less drastic for inoculated plants. For chlorophyll a, the decrease was by 19.4% in AMF-inoculated plants compared to 28.8% in non-inoculated plants. For total chlorophylls, the reduction was by 19.8% in inoculated plants and 28.1% in non-inoculated plants. Regarding chlorophyll b contents, (MI)_1_ and (MI)_2_ allowed a decrease by 24 and 14%, respectively, compared to 24.5% in non-inoculated plants. For carotenoids, water stress induced an increase in their contents for inoculated and non-inoculated plants (Figure 4D). When comparing inoculated plants to non-inoculated ones, carotenoid contents were generally higher by 23.6 and 16.2% under WW and DS conditions, respectively. Additionally, mycorrhizal inoculation allowed an increase of 29.6% of its contents.

### 3.5. Malonaldehyde Content (MDA)

**Figure 5:**
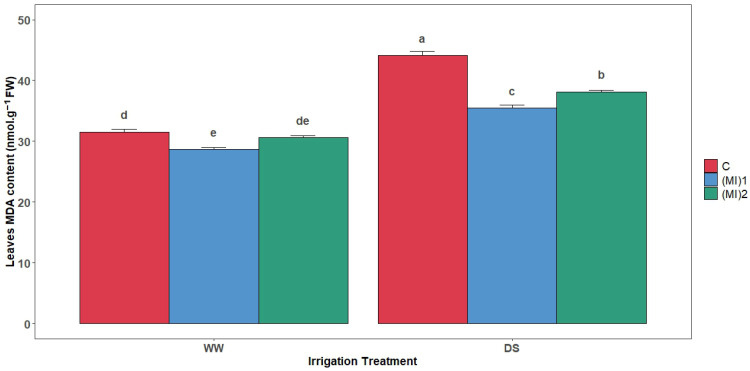
Malonaldehyde content (MDA) in leaves of potato (*Solanum tuberosum* L.) plants non-inoculated (control: C) or inoculated with the two mycorrhizal inocula (MI)_1_ and (MI)_2_ and cultivated under well-watered (WW) and drought stress (DS) conditions. Different letters indicate significant differences between all treatments according to two-way ANOVA followed by Tukey’s test (*p* < 0.05).

Drought stress significantly increased MDA content in potato leaves (Figure 5). However, the results show that AMF inoculation reduced MDA content under both WW and DS conditions. In fact, inoculation with (MI)_1_ and (MI)_2_ induced a decrease in leaf MDA content by 9.2% and 2.9%, respectively, under WW conditions, and by 19.5% and 13.4% under DS conditions, compared to control plants.

### 3.6. Antioxidant Enzymatic Activities

**Figure 6:**
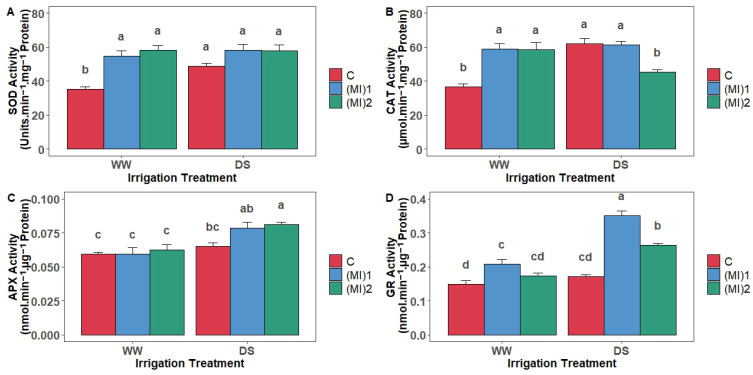
Antioxidant enzyme activities in leaves of potato (*Solanum tuberosum* L.) plants non-inoculated (control: C) or inoculated with the two mycorrhizal inocula (MI)_1_ and (MI)_2_ and cultivated under well-watered (WW) and drought stress (DS) conditions; (**A**) superoxide dismutase (SOD), (**B**) catalase (CAT), (**C**) ascorbate peroxidase (APX), and (**D**) glutathione reductase (GR). Different letters indicate significant differences between all treatments according to two-way ANOVA followed by Tukey’s test (*p* < 0.05).

Water stress did not affect the SOD activity of AMF-inoculated plants, but this activity increased by 38.9% in non-inoculated plants. Additionally, under WW conditions, mycorrhizal inoculation allowed a significant increase in CAT activity. In contrast, under DS conditions, there was an increase in CAT activity by 68.7% in non-inoculated plants and a decrease by 23% in plants inoculated with (MI)_2_. APX activity increased significantly under DS conditions, especially in plants inoculated with (MI)_2_. This increase was by 23.5% in AMF-inoculated plants, while non-inoculated plants showed a significantly lower increase of 8.7%. Furthermore, water stress induced a highly significant increase in GR activity in AMF-inoculated plants, with the highest values reaching 0.351 nmol/min/µg protein in plants inoculated with (MI)_1_. This increase was approximately by 68 and 52% under water stress conditions compared to WW conditions for (MI)_1_ and (MI)_2_, respectively (Figure 6).

### 3.7. Antioxidant Enzymes Gene Expression Levels

**Figure 7:**
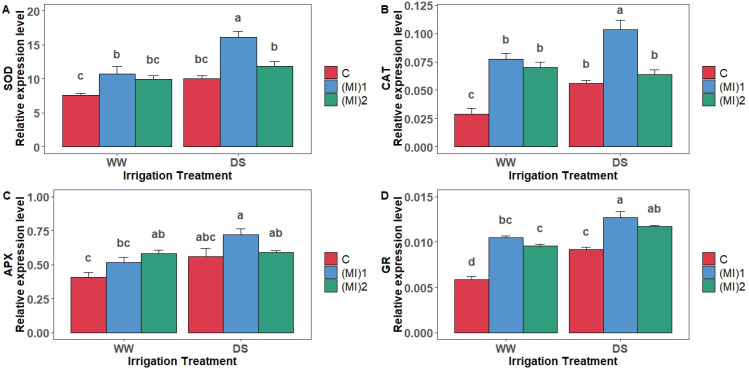
Relative expression level of (**A**) superoxide dismutase (SOD), (**B**) catalase (CAT), (**C**) ascorbate peroxidase (APX), and (**D**) glutathione reductase (GR) genes in potato (*Solanum tuberosum* L.) plants non-inoculated (control: C) or inoculated with the two mycorrhizal inocula (MI)_1_ and (MI)_2_ and cultivated under well-watered (WW) and drought stress (DS) conditions. Different letters indicate significant differences between all treatments according to two-way ANOVA followed by Tukey’s test (*p* < 0.05).

Our results indicated that drought stress significantly upregulated the expression of one SOD gene in potato leaves inoculated with (MI)1, resulting in a 50.5% increase (Figure 7A).

CAT gene expression was strongly induced by mycorrhizal inoculation under WW conditions, with increases of 140.8% in plants inoculated with (MI)1 and 93.5% with (MI)2. Under DS conditions, a significant increase in CAT expression was observed only in plants inoculated with (MI)1, showing an upregulation of 38.4% (Figure 7B).

APX gene expression increased in response to AMF inoculation under both WW and DS conditions. Under WW conditions, APX expression increased by 26.8% with (MI)1 and by 42.5% with (MI)2 compared with non-inoculated plants (Figure 7C). Under DS conditions, APX expression was upregulated by approximately 40% in plants inoculated with (MI)1.

GR gene expression was markedly induced by AMF inoculation under both watering regimes (Figure 7D). Under WW conditions, GR expression increased by 78.6% with (MI)1 and by 62.4% with (MI)2 relative to non-inoculated plants. Under DS conditions, GR expression increased by 38.5% and 28.7% with (MI)1 and (MI)2, respectively. In addition, drought stress further enhanced GR expression by 21% with (MI)1 and by 23.6% with (MI)2 (Figure 7).

### 3.8. Pearson’s Correlation Analysis

**Figure 8:**
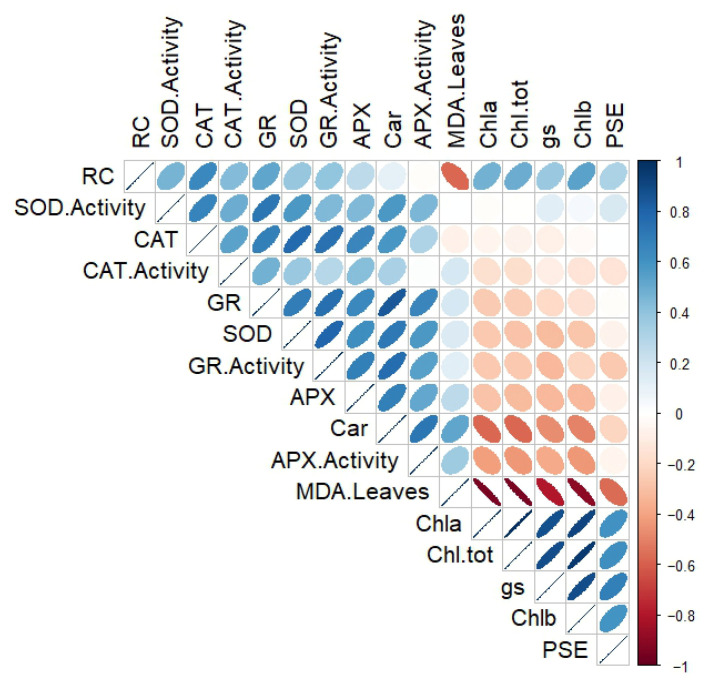
Pearson’s correlation matrix between physiological and biochemical parameters measured in potato (*Solanum tuberosum* L.) plants. Color gradient 0 to 1 indicates a positive correlation and 0 to −1 indicates a negative correlation between the studied parameters. RC: root colonization, PSE: photosynthetic efficiency, gs: stomatal conductance, Chla: chlorophyll a, Chlb: chlorophyll b, Chl-tot: total chlorophylls, Car: carotenoids, MDA leaves: malondialdehyde content in leaves, SOD activity: superoxide dismutase activity, CAT activity: catalase activity, APX activity: ascorbate peroxidase, GR activity: glutathione reductase, SOD: superoxide dismutase gene expression, CAT: catalase gene expression, APX: ascorbate peroxidase gene expression, and GR: glutathione reductase gene expression.

The analysis revealed that mycorrhizal root colonization (RC) was positively correlated to photosynthetic efficiency (PSE), stomatal conductance (gs), photosynthetic pigments contents (Chla, Chlb, Chl-tot, and Car), the antioxidant enzymatic activities, and their gene expression levels. No significant correlation was found between RC and APX activity. Conversely, RC was negatively correlated to malondialdehyde (MDA) content in leaves. MDA content showed high negative correlations with PSE, gs, and photosynthetic pigments contents, but a positive correlation with all measured antioxidant enzymatic activities, except SOD. A positive correlation was found between MDA content and the gene expression levels of all studied antioxidant enzymatic activities, except CAT. Moreover, CAT, GR, and APX activities and their gene expression levels were negatively correlated to PSE, gs, and photosynthetic pigments contents (Figure 8).

### 3.9. Principal Component Analysis (PCA)

**Figure 9:**
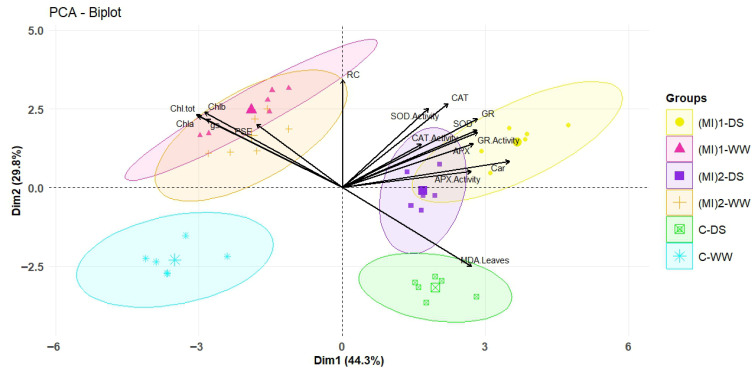
Principal component analysis (PCA) biplot of physiological and biochemical parameters measured in potato (*Solanum tuberosum* L.) plants non-inoculated (control: C) or inoculated with the two mycorrhizal inocula (MI)_1_ and (MI)_2_, and cultivated under well-watered (WW) and drought stress (DS) conditions. RC: root colonization, PSE: photosynthetic efficiency, gs: stomatal conductance, Chla: chlorophyll a, Chlb: chlorophyll b, Chl-tot: total chlorophylls, Car; carotenoids, MDA leaves: malondialdehyde content in leaves, SOD activity: superoxide dismutase activity, CAT activity: catalase activity, APX activity: ascorbate peroxidase, GR activity: glutathione reductase, SOD: superoxide dismutase gene expression, CAT: catalase gene expression, APX: ascorbate peroxidase gene expression, and GR: glutathione reductase gene expression.

The combined principal components PC1 and PC2 (Dim1 and Dim 2) account for 74.1% of the variance, indicating a strong association among the studied parameters (Figure 9). The results revealed an opposing effect between non-inoculated (control) and inoculated treatments, under both well-watered and drought stress conditions. All photosynthetic activity parameters (Chla, Chlb, Chl-tot contents, PSE, and gs) were highly associated with AMF inoculation under well-watered conditions. The highest carotenoids content and antioxidant enzyme activities and their gene expressions were linked to AMF inoculation under water stress conditions. However, MDA was highly associated with non-inoculated plants under water stress conditions.

## 4. Discussion

Drought remains one of the most detrimental abiotic stresses affecting potato (*S. tuberosum* L.) productivity [6,7]. Insufficient water disrupts physiological and biochemical processes, impairing tuber yield and biomass accumulation [41]. Although research on potato drought tolerance is still limited, interest has grown as the crop expands into semi-arid and arid regions [42,43]. Concurrently, AMF have gained attention for their ability to enhance plant tolerance to environmental stresses [18,44,45]. This study highlights the multifaceted contribution of AMF inoculation to drought mitigation in potato through combined physiological and biochemical mechanisms.

Variations in mycorrhizal colonization under different irrigation regimes suggest a dynamic interaction between AMF and roots, influencing water and nutrient uptake. The significant reduction in AMF colonization under drought aligns with previous studies showing that water limitation negatively affects fungal growth, likely due to decreased spore germination and restricted hyphal proliferation [26,46,47,48,49]. Under well-watered conditions, AMF inoculation increased shoot biomass, reflecting enhanced water and nutrient acquisition. AMF improve phosphorus uptake, water balance via increased root hydraulic conductivity, and root morphology, with fungal hyphae expanding soil volume explored by roots [50,51,52]. This synergistic relationship enhances nutrient use efficiency and aligns with previous biomass increases observed under optimal conditions [53,54]. However, under drought, biomass enhancement was limited, indicating that while AMF alleviate stress, growth-promoting effects are constrained under severe water deficit [18,55].

AMF symbiosis tended to increase total pigment content and stomatal conductance (gs), while no consistent change in PSII photochemical efficiency was detected. This may be due to the conserved structural organization of the PSII core complex and light-harvesting pigments [56], limiting detectable differences. Nevertheless, the physiological stability suggests improved water use efficiency and sustained photosynthesis, crucial under drought. Previous studies similarly reported that AMF sustain photosynthetic rates and modulate stomatal behavior under water deficit [18,57,58]. AMF improved leaf gas exchange and preserved PSII efficiency under drought [59]. Moreover, AMF inoculation significantly increased chlorophyll a, chlorophyll b, total chlorophyll, and carotenoids under both well-watered and drought conditions, supporting enhanced pigment synthesis under stress, as observed in maize [60] and trifoliate orange [26]. Enhanced chlorophyll may maintain photosynthetic capacity [61,62], while carotenoids provide photoprotection by quenching triplet chlorophyll and scavenging singlet oxygen, reducing photooxidative damage [35,63].

Under drought, decreased MDA in AMF-inoculated plants indicated reduced lipid peroxidation and ROS damage [26,49]. Variations in antioxidant enzyme activities further elucidate AMF-mediated drought tolerance. SOD activity remained stable in AMF-inoculated plants under drought, unlike the pronounced increase in non-inoculated plants, reflecting a more efficient redox balance, similar to findings in tea seedlings [25]. CAT activity increased under well-watered conditions with AMF, but decreased under drought in some inoculated plants, while non-inoculated plants showed increased CAT under stress. APX activity rose under drought, particularly in AMF-inoculated plants, highlighting activation of the ascorbate–glutathione pathway [48]. GR activity increased in all AMF-inoculated plants under drought, supporting ROS detoxification [64,65,66]. Gene expression patterns mirrored enzymatic activities: SOD and CAT expression increased under AMF inoculation, APX and GR expression were significantly higher in inoculated plants under both conditions, confirming AMF stimulation of ROS detoxification at the transcriptional level [25,66,67].

AM symbiosis may also influence root metabolites. Hu et al. [68] reported increased primary metabolites (fatty acids, organic acids, polyamines) under drought in AMF plants. Plant hormones regulated by AMF could further enhance antioxidant defense [69].

Differential responses between the two AMF inocula likely reflect inoculum-specific traits, ecological origin, and adaptation to environmental conditions. Although both belong to Rhizophagus irregularis, MI1 (Spain) may be more compatible with northern Mediterranean environments, whereas MI2 (Tunisia) may perform better in arid/semi-arid zones. Functional differences may also reflect host genotype interactions, emphasizing the importance of selecting AMF strains based on environmental origin, ecological adaptation, and host compatibility [70,71,72].

In summary, AMF inoculation enhances potato antioxidant defense by modulating enzyme activities and gene expression, improving drought tolerance. Strong correlations between root colonization and physiological/biochemical traits, including photosynthetic efficiency, gs, pigment content, and antioxidant activity, highlight the systemic integration of AMF-mediated responses [26,73]. PCA clearly distinguished inoculated from non-inoculated plants under both conditions, demonstrating the consistent and comprehensive impact of AMF on plant function and supporting its potential as a sustainable strategy to enhance drought resilience.

## 5. Conclusions

Overall, AMF-inoculated potato plants exhibited enhanced drought tolerance compared to non-inoculated controls, as reflected by improved photosynthetic efficiency, greater stomatal conductance, and increased photosynthetic pigment contents. The study also revealed significant differences in leaves’ MDA contents and antioxidant enzymes and their gene expression levels, which varied according to both inoculum type and water regimes. Additionally, these findings underscore the complex and inoculum-specific nature of AMF–plant interactions under water stress conditions. Future research should focus on exploring the molecular pathways through which each AMF strain modulates drought tolerance, with particular attention to host genotype compatibility. Finally, investigating the variability of these mechanisms across different potato cultivars will be essential to optimize AMF-based strategies for sustainable crop production in diverse agroecological contexts.

## Figures and Tables

**Table 1:** Primer sequences of genes used for RT–PCR analysis.

Name	NCBI Acc. No.	Primer Sequence (5’–3’)
Actin	X55749	F-CTGGTGGTGCAACAACCTTA R-GAATGGAAGCAGCTGGAATC
APX	AB041343	F-ACCAATTGGCTGGTGTTGTT R-TCACAAACACGTCCCTCAAA
CAT	AY442179	F-TGCCCTTCTATTGTGGTTCC R-GATGAGCACACTTTGGAGGA
GR	X76533	F-GGATCCTCATACGGTGGATG R-TTAGGCTTCGTTGGCAAATC
SOD	AF354748	F-GTTTGTGGCACCATCCTCTT R-GTGGTCCTGTTGACATGCAG

## Data Availability

Data will be provided upon request.

## Abbreviations

The following abbreviations are used in this manuscript:

AMF	Arbuscular Mycorrhizal Fungi
AsA	Ascorbic Acid
PSE	Photosystem II efficiency
gs	Stomatal conductance
GSH	Glutathione
MDA	Malondialdehyde
SOD	Superoxide Dismutase
CAT	Catalase
APX	Ascorbate Peroxidase
GR	Glutathione Reductase
WW	Well-watered
DS	Drought stress
RC	Root colonization
ROS	Reactive Oxygen Species
(^1^O_2_)	singlet oxygen
(H_2_O_2_)	hydrogen peroxide
Chla	Chlorophyll a
Chlb	Chlorophyll b
Chl-tot	total chlorophylls
Car	Carotenoids
